# Investigating factors influencing utilization of trauma-focused cognitive behavioral therapy among unaccompanied young refugees: an exploratory analysis

**DOI:** 10.1186/s13034-025-00862-z

**Published:** 2025-02-06

**Authors:** Barbara Kasparik, Madina Farani, Elisa Pfeiffer, Cedric Sachser, Rita Rosner

**Affiliations:** 1 Department of Psychology, Catholic University Eichstätt-Ingolstadt, Ostenstraße 25, 85072 Eichstätt, Germany; 2 Department of Child and Adolescent Psychiatry/Psychotherapy, Ulm University, Ulm, Germany; 3 Partner site Ulm, German Center for Mental Health (DZPG), Ulm, Germany; 4 Department of Clinical Child and Adolescent Psychology, Otto Friedrich University of Bamberg, Kapuzinerstraße 32, 96047 Bamberg, Germany

**Keywords:** Unaccompanied young refugees, Asylum seekers, Mental health care, PTSD, TF-CBT, Help seeking intention, Utilization

## Abstract

**Background:**

Unaccompanied young refugees (UYRs) exhibit elevated levels of mental distress, including posttraumatic stress symptoms (PTSS), depression and anxiety. Despite the considerable psychological burden, UYRs frequently lack access to mental health care (MHC). The factors that contribute to higher rates of treatment utilization among UYR remain poorly understood. Untreated PTSS can result in chronic impairment, underscoring the importance of identifying these factors. The aim of this study is to investigate factors associated with the intention and actual utilization of MHC of UYRs living in child and youth welfare facilities in Germany.

**Method:**

This study is part of the multi-site project BETTER CARE which aims to implement a stepped and collaborative care approach. A sample of *N* = 139 UYRs who had received a treatment recommendation for trauma-focused cognitive behavioral therapy (TF-CBT) was analyzed. Binomial logistic regression was performed to identify factors predicting the likelihood of intention to seek MHC. In addition, the association between intention to seek MHC and actual utilization was determined using a chi square test.

**Results:**

The results demonstrated a significant correlation between age (*η* = 0.25, *p* <.01), length of stay in Germany (*η* = 0.28, *p* <.01), and severity of PTSD symptoms (*η* = 0.26, *p* <.01) with intention to use MHC. In the logistic regression analysis, PTSD emerged as a significant predictor of intended use (B = 2.66, *p* <.05). The utilization of MHC was found to be closely associated with the initial intention to use (*χ*²(1) = 88.846, *p* <.001).

**Conclusions:**

The findings contribute to an expanding body of literature on the mental health requirements and service utilization patterns among UYRs, offering insights for policymakers, mental health professionals, and child welfare services striving to enhance care for this vulnerable population.

**Supplementary Information:**

The online version contains supplementary material available at 10.1186/s13034-025-00862-z.

## Background

Unaccompanied young refugees (UYRs), who are children, adolescents or young adults who flee their home country unaccompanied by parents or other caregivers, often experience stressful and traumatic events in their home countries, during their flight and upon arrival in their host country [1]. They also face post-migration challenges such as discrimination and insecure living conditions [2, 3]. Experiencing potentially traumatic events, facing post-migration challenges, and encountering various psychological stressors can heighten the likelihood of developing common mental health disorders, particularly post-traumatic stress disorder (PTSD), anxiety, or depression [2, 4–6]. Prevalence rates of mental disorders in this population vary widely across studies, ranging from 4.6 to 43% for PTSD, 2.9–61.6% for depression, 32.6–38.2% for anxiety, and 4–14.3% for behavioral problems [7]. In addition to high prevalence rates, UYRs often lack protective factors compared to other immigrant groups [3]. Separation from significant others (e.g., parents) and lack of social support may result in increased psychopathology among UYRs compared to native and refugee youth with parental caregivers [8–10]. Additionally, UYRs experience significantly more stressful life events than accompanied young refugees, which is a significant predictor of symptoms related to posttraumatic stress (PTSS), as well as depression and anxiety [9,11].

A variety of treatment approaches are available for the treatment of PTSD in children and adolescents and for young refugees. Of these approaches, trauma-focused cognitive behavioral therapy (TF-CBT) is the most thoroughly researched and most well-documented intervention in terms of effectiveness [12]. Systematic reviews, e.g. by Xiang et al. [13] and Thielemann et al. [14], have demonstrated the effectiveness of a specific CBT-based trauma-focused manual, as developed and described by Cohen, Mannarino, and Deblinger [15, 16]. In addition, TF-CBT has been endorsed by international guidelines for the treatment of PTSD in children and adolescents [17]. TF-CBT is a culturally sensitive approach that is capable of addressing the individual cultural needs of patients [16, 18]. It has also been demonstrated to be efficacious in non-Western countries [19, 20] and in refugee populations [21, 22]. Research has demonstrated that access to mental health care is a significant predictor of improvement of PTSS, e.g [23]. Furthermore, untreated PTSD can result in chronic impairment [24]. Despite the existence of evidence-based treatments (EBT), a significant proportion of refugees experiencing mental health issues, particularly PTSD, are not adequately screened and fail to receive adequate mental health care (MHC) [25, 26]. It is often the case that structural barriers, including lacking financial resources and a lack of language skills, impede refugees from accessing MHC [27, 28]. The situation of UYRs is more pronounced due to their low utilization of MHC and lack of access to specific EBTs, resulting in higher unmet needs compared to resident peers and accompanied young refugees [8, 29–32]. In the absence of appropriate treatment of PTSS, long-term impairment may ensue [24]. This can result in personal distress and may also impede the integration of UYR, thereby hindering their successful resettlement in the host country [33, 34].

Given that not all young refugees with PTSS and associated distress receive adequate treatment, it is crucial to identify factors associated with help-seeking behavior and utilization of MHC. In recent years, a number of theories have been used to explain help-seeking and the use of MHC, one of which is Ajzen’s *Theory of Planned Behavior* (TPB) [35, 36]. It posits that individuals deliberately opt to engage or not engage in certain behaviors, influenced by their *attitudes*, perceived social pressures (*subjective norms*), and beliefs about their capacity to control these behaviors (*perceived behavioral control*). These components are designed to predict the intention that will lead to the performance of the behavior [36]. The intention to seek help for mental health problems is a representation of willingness. It is a deliberate act of communication with external sources, and, in conjunction with perceived behavioral control, it is a predictor of the actual utilization of MHC [37]. A number of meta-analyses and reviews have demonstrated the efficacy of the TPB in the context of health behaviors [35; 38].

The extant literature addresses several factors that have been identified as contributing to increased utilization of MHC, though the findings are not entirely consistent. Given the small data bases on the utilization of MHC of UYRs, it is necessary reviewing the literature on utilization behavior in adjacent populations sharing relevant characteristics with UYRs, such as adult refugees, immigrants and the broader population of adolescents and young adults. In the context of adolescents and young adults without a refugee or migration background, several factors have been identified as potential contributors to increased utilization of MHC: externalizing behavior, overall problem level, delinquent behavior, and impairment [39]. Furthermore, positive previous experiences with MHC, mental health literacy, and a strong bond with the caregiver were identified as contributing factors to utilization of MHC [40]. Adolescents may be deterred from utilizing MHC due to concerns about stigmatization, heightened symptomatology, and negative perceptions of both MHC and the professionals who provide them [40]. The impact of gender, socioeconomic status, and ethnic background remains inconclusive, with findings on these factors exhibiting inconsistency [39].

Some of the factors associated with the use of MHC by immigrants and refugees have already been identified in research on the general population. However, additional relevant factors were identified for this specific population. In addition to an increased severity of PTSS, awareness of mental health and mental health literacy [41], a higher level of education, female gender, insecure asylum status, and the length of stay in the host country are associated with increased utilization of MHC among adult refugees and immigrants [23, 25, 27, 28, 41–43]. Individuals with poor general health status are more likely to utilize MHC services [42], though refugees and migrants tend to seek assistance predominantly from medical rather than psychotherapeutic services [44, 45]. Other reasons that can lead to refugees not seeking MHC include a lack of recognition of their own need for such care compared to the need of family members, fear of being rejected by family or acquaintances, and the attribution of mental health issues to supernatural causes [46–48].

The existing literature addresses several key elements that contribute to the utilization of MHC by UYRs. To date, the evidence on factors that act as barriers or facilitators to the utilization of MHC by UYRs is limited. The following factors have already been identified as relevant: the length of time spent in the host country, the number of traumatic events experienced, the severity of symptoms, younger age, the availability of help-seeking support from caregivers as well as the self-reported need for MHC [8, 29, 30, 32]. Conversely, the presence of depressive symptoms may lead to lower rates of MHC use [32]. UYRs often view symptoms as an inevitable outcome of traumatic experiences, which can also hinder the utilization of MHC [32, 48].

## Aim of the study

In light of the limited and inconsistent research on the use of MHC in general and by UYRs in particular, our study seeks to identify factors associated with intended and actual use of MHC by UYRs. Given the existence of effective treatment options and the potential negative consequences of untreated PTSD, it is crucial to identify barriers and facilitators to MHC in order to provide further support to this specific group. The objective of this exploratory study is to examine the influence of sociodemographic variables and symptom scores (PTSD, depression, anxiety) on the intention to utilize MHC, as well as the association between intention and actual utilization of MHC in a sample of UYRs.

The following research questions guided the current study:

- What factors (sociodemographic factors, symptom severity, probable comorbid diagnoses) are associated with intent to utilize MHC?
- What factors (sociodemographic factors, symptom severity, probable comorbid diagnoses) are associated with actual MHC utilization?
- Are there any factors that may serve as predictors of the intention to utilize MHC?
- Does the intention to utilize MHC lead to actual MHC utilization?

## Method

### Sample and procedure

The data were collected as part of the multi-center, cluster-randomized controlled trial BETTER CARE [49]. The project was approved by the ethics committees at Ulm University (No. 243/19) and at Catholic University of Eichstätt-Ingolstadt (No. 004–19). The BETTER CARE project was designed to implement and examine a screen and stepped-care approach for UYRs providing empirically supported treatment. A total of 627 UYRs living in 58 child and youth welfare service (CYWS) facilities participated in the screening of UYRs between July 2020 and July 2024. The advent of the COVID-19 pandemic in 2020 necessitated the implementation of daily life restrictions, which consequently impacted data collection procedures. Consequently, assessments were conducted online or on-site, in accordance with established hygiene protocols. As part of the study, participants were compensated with 30-euro vouchers for each assessment. There was no compensation for participation in psychotherapy sessions. Following the initial assessment (T0), all UYRs residing in a participating CYWS facility were randomly allocated to either the Better Care (BC) condition or a control condition designated as Usual Care + (UC+). Twenty-nine CYWS facilities were randomly assigned both to the BC and the UC + condition.

The stepped-care approach commenced with the initial step, which entailed screening and treatment recommendation. The second step involved enrolling subclinical cases in the preventive group program “Mein Weg” [50]. UYRs above the clinical cut-off (Child and Adolescent Trauma Screen, CATS-2 ≥ 25) were referred to the third step, TF-CBT as defined by Cohen et al. [15; 16]. The present study examined a subsample of UYRs who received a treatment recommendation for TF-CBT. The final sample of UYRs with treatment recommendation was *N* = 139 UYRs. Of these, *n* = 18 were excluded from the analysis as they had already undergone psychotherapy at the time of our treatment offer, *n* = 14 were excluded due to relocation, and *n* = 2 were excluded due to the initiation of inpatient psychiatric treatment. For UYRs with a TF-CBT referral, following the initial screening, caregivers from the CYWS facility were queried as to the UYRs intention to pursue therapy. If this was the case, the study staff established contact with a collaborating psychotherapist in the vicinity of the child and youth welfare service (CYWS) facility. For both study arms, follow-up screenings took place 6 (T1) and 12 (T2) months after the initial screening. Inclusion criteria for participants were: (1) age between 12 and 20 years, (2) arrival in Germany as an unaccompanied minor, (3) application for asylum or intent to do so, (4) being cared for by a CYWS facility, (5) written informed consent by the participant and legal guardian (if under 16 years), and (6) report of at least one traumatic event according to the DSM-5 A criterion. No exclusion criteria have been defined.

### Measures

Questionnaires were provided in multiple languages, including German, English, French, Arabic, Dari, Farsi, Pashto, Somali, Tigrinya, Russian, Ukrainian, and Kurmanci. Should the need arise, interpreters were available in person or by telephone. The demographic data collected encompassed a range of variables, including age, gender, religious beliefs, length of stay in Germany and within the CYWS premises, current school attendance, and residence status. The UYR’s caregivers were inquired about the intention of the UYR to utilize MHC and this information was meticulously recorded. The actual utilization of MHC was documented by the treating study psychotherapists.

#### Posttraumatic stress symptoms

The assessment of post-traumatic stress symptoms (PTSS) in children and adolescents was conducted using the *Child and Adolescent Trauma Screen* (CATS-2) by Sachser et al. [51]. The questionnaire employs a 15-item checklist to identify potential traumatic events (PTEs) and subsequently measures the severity of PTSS through a 20-item scale, with responses collected on a 4-point Likert scale. The overall DSM-5 PTSS severity is determined by adding the scores from items 1 to 20 (with a possible range of 0 to 60), incorporating only the highest score from items 9, 10, and 15. The cut-off score for clinically relevant PTSD symptoms is 25. The CATS-2 is available in two versions, one for self-reporting and one for caregiver-reporting. In our study, the instrument demonstrated good internal consistency, achieving Cronbach’s *α* scores of 0.76.

#### Depressive symptoms

The *Patient Health Questionnaire* (PHQ-9) is a measurement tool comprising nine items that correspond to the Diagnostic and Statistical Manual of Mental Disorders (DSM-IV) criteria for detecting depressive symptoms. Responses are collected using a four-point Likert scale. The instrument exhibited robust internal consistency, as evidenced by a Cronbach’s alpha score of 0.76. The instrument has been validated in diverse settings and languages [52].

#### Anxiety symptoms

The *Generalized Anxiety Disorder Assessment* (GAD-7) is a seven-item instrument that evaluates anxiety symptoms on a four-point Likert scale, based on the Diagnostic and Statistical Manual of Mental Disorders (DSM-IV) criteria. The instrument demonstrated high internal consistency (*α* = 0.82) and has been validated in numerous contexts and languages [52].

### Data analysis

Analyses were conducted using IBM SPSS Statistics, version 29. A significance level of *p* <.05 (two-tailed) was predetermined for all analyses. Given the exploratory nature of this analysis, the initial step was to construct correlation coefficients to ascertain the relationships between the sociodemographic variables and mental health parameters and the intention and actual utilization of MHC. We operationalized the likelihood of depression or anxiety comorbidity with PTSD, considering a comorbid disorder likely to be present if either the PHQ-9 or the GAD-7 score was above the cutoff for clinically relevant symptomatology, and also calculated the association between probable comorbidity and intention and MHC utilization.

A binomial logistic regression was performed to determine the effect of age, length of stay in Germany and PTSD symptom severity in predicting the likelihood of intending to use MHC. The linearity of the data was evaluated using the Box-Tidwell procedure [53]. A Bonferroni correction was applied to all terms in the model. All variables were found to have a linear relationship. Multicollinearity was tested but did not significantly affect the analysis. The data set revealed no cases that could be considered potential outliers. Sensitivity analyses were performed based on the cut-off scores for clinically relevant depressive and anxiety symptoms. Regression analyses were conducted in four distinct groups: (a) UYRs above the cut-off for clinically relevant symptoms in the PHQ-9, (b) UYRs below the cut-off for clinically relevant symptoms in the PHQ-9, (c) UYRs above the cut-off for clinically relevant symptoms in the GAD-7, and (d) UYRs below the cut-off for clinically relevant symptoms in the GAD-7. To examine the association between intention and utilization of MHC, a chi square test was conducted, using Fisher’s exact probability test to calculate statistical significance.

## Results

### Sample characteristics

Sociodemographic characteristics are summarized in Table 1. The average age of the sample was 16.72 years (*SD* = 1.35), with ages ranging from 13 to 20 years. The majority of participants were male (87%) and identified as Muslim (91%). Most participants were of Afghan (51.1%) or Syrian (13.7%) background. Participants had an average length of stay in Germany of 15.68 months (*SD* = 18.69, range 0–95). Given their relatively young age, the majority of participants (70%) reported a relatively secure status with regard to their residence, either in the form of a temporary residence permit or permission to stay. The average number of potentially traumatic events (PTEs) reported by UYR was 8, with a range of 1 to 14 events. Symptom scores indicated a clinically significant level of distress for PTSD, depression, and anxiety, with average scores of 35.99 (CATS-2, *SD* = 7.62), 13.61 (PHQ-9, *SD* = 5.52), and 11.58 (GAD-7, *SD* = 4.79) respectively. All UYRs exhibited clinically significant PTSS based on the established inclusion criteria. Regarding depressive symptoms, *n* = 110 UYRs (79%) demonstrated a clinically significant symptom level, while *n* = 80 UYRs (58%) exhibited clinically significant symptoms of anxiety.

**Table 1:** Descriptive characteristics of participating UYR

	*n*	%	M (SD); range
Age	139		16.72 (1.35); 13–20
Gender			
Male	121	87.1	
Female	17	12.2	
Diverse	1	0.7	
Country of origin			
Afghanistan	71	51.1	
Syria	19	13.7	
Somalia	8	5.8	
Iraq	6	4.3	
Other^1^	35	25.1	
Religion			
Muslim	127	91.4	
Christian	4	2.9	
Other^2^	2	1.4	
Non-religious	6	4.3	
Residence status			
Secure residence status^3^	93	66.9	
Uncertain residence status^4^	29	27.1	
Length of stay in Germany (months)	137		15.68 (18.69), 0–95
Current school attendance	122	87.8	
Number of PTEs	139		8.10 (2.97), 1–14
CATS-2	139		35.99 (7.62), 25–56
PHQ-9	139		13.61 (5.52), 0–27
GAD-7	139		11.58 (4.79), 0–21

^(1)^ Albania, Cameroon, Eritrea, Gambia, Guinea, Iran, Libya, Mali, Marocco, Nigeria, Pakistan, Rumania, Sierra Leone, Tunisia, Turkey; ^(2)^ Buddhism, Hinduism, Judaism; ^(3)^ Temporary residence permit, pending process; ^4^ Negative decision, tolerated stay, other; CATS-2 Child and Adolescent Trauma Screen 2; PHQ-9 Patient Health Questionnaire-9; GAD-7 Generalized Anxiety Disorder Scale-7

### Factors predicting the intention and utilization of MHC

A preliminary investigation was undertaken to ascertain the potential factors that may influence intention and utilization of MHC. Exploratory, we calculated correlation coefficients. As shown in Table 2, age (*η* = 0.25, *p* <.01), length of stay (*η* = 0.28, *p* <.01), and PTSD symptom severity (*η* = 0.26, *p* <.01) were significantly correlated with intention to use MHC. Only intention to use MHC correlated significantly with actual utilization (*φ* = 0.92, *p* <.001). Socio-demographic factors, symptom severity and probable presence of a comorbid diagnosis were not significantly associated with utilization.

**Table 2:** Correlations between predictors and intention/utilization of MHC

Variable	Intention to utilize MHC		Utilization of MHC	
	Correlation coefficient	Coefficient	Correlation coefficient	Coefficient
Age	Eta	0.25**	Eta	0.23
Gender	Phi	0.02	Phi	0.05
Region of origin	Contingency coefficient	0.17	Contingency coefficient	0.17
Religion	Contingency coefficient	0.21	Contingency coefficient	0.19
Residence status	Phi	0.07	Phi	0.13
Length of stay	Eta	0.28**	Eta	0.25
Current school attendance	Phi	0.13	Phi	−0 0.08
Number of PTEs	Eta	0.11	Eta	0.10
CATS-2	Eta	0.26**	Eta	0.24
Reexperiencing	Eta	0.26**	Eta	0.36
Avoidance	Eta	0.13	Eta	0.29
Cognition & Mood	Eta	0.05	Eta	0.35
Hyperarousal	Eta	0.21*	Eta	0.42
PHQ-9	Eta	0.10	Eta	0.13
GAD-7	Eta	0.02	Eta	0.06
Probable comorbidity	Phi	0.03	Phi	0.19
Intention	–	–	Phi	0.92***

* n* = 125 − 106, CATS-2 Child and Adolescent Trauma Screen 2, PHQ-9 Patient Health Questionnaire-9, GAD-7 Generalized Anxiety Disorder Scale-7; *p <.05, **p <.01, ***p <.001

The binomial logistic regression model on the intention to utilize MHC was statistically significant, *χ*²(3) = 13.19, *p* <.05, with an amount of explained variance as shown by Nagelkerke’s *R*²= 0.17. Overall percentage of accuracy in classification was 72%. Of the three variables entered into the regression model, the CATS-2 score contributed significantly to the prediction of intention to use MHC with *b* = 2.66 (*p* <.05). Higher CATS-2 scores increase the likelihood of intending to use MHC with OR = 14.32 (95% CI [1.37, 149.56]). All model coefficients and odds can be found in Table 3. The sensitivity analyses demonstrated that factors which significantly contributed to the prediction of the intention to utilize MHC were not influenced by the presence or absence of clinically relevant depressive or anxiety symptoms (see Supplement, Table S1).

**Table 3:** Results of the binomial logistic regression analysis: intention to utilize MHC (n = 105)

						95% CI for OR	
	B	SE	Wald	*p*	OR	Lower	Upper
Age	0.23	0.19	1.51	0.220	0.79	0.54	1.15
Length of stay	-0.38	0.28	1.83	0.176	0.69	0.40	1.19
CATS-2	2.66	1.20	4.95	0.026	14.32	1.37	149.56
Constant	-3.88	5.05	0.59	0.442	0.02		

CATS-2 Child and Adolescent Trauma Screen 2, *R*^2^ = 0.12 (Cox & Snell), 0.17 (Nagelkerke), Model *χ*^2^ (3) = 13.19, *p* <.05

To investigate the association between intention and actual utilization, a chi square test was conducted, using Fisher’s exact probability test to calculate statistical significance. As shown in Tables 4 and 63% of the UYR who had the intention to utilize MHC did, in fact, utilize MHC. In our sample, no UYR exhibited a lack of intention and yet utilized MHC. Fisher’s exact test indicated a statistically significant association between the intention and actual utilization (*p* <.001).

**Table 4:** Frequencies and percentages of intention and utilization

	No utilization of MHC	MHC utilization
No intention to utilize MHC	35 (33.33%)	0 (0%)
Intention to utilize MHC	4 (3.8%)	66 (62.86%)

## Discussion

Existing literature has shown that UYRs are a particularly vulnerable sample with high rates of psychological distress. Despite this knowledge, there is a significant gap in the existing literature regarding the correlates of utilization of MHC in this population. This study examined the influence of sociodemographic variables and symptom severity (PTSD, depression, anxiety) on the intention to utilize MHC, as well as the association between intention and actual utilization in a sample of UYRs. We found significant associations between age, length of stay, PTSS and intention to seek MHC. The actual utilization of MHC was only related to the initial intention to do so. No associations were found between the number of PTEs experienced and intention or utilization. There were also no associations between depressive symptoms and intention or utilization. The regression analysis demonstrated that only PTSS was ultimately a significant predictor of intention to use MHC. In line with existing literature, age and length of stay in the country of resettlement are factors that contribute to an increased utilization of MHC services [29, 32]. Despite the absence of a statistically significant contribution of these predictors in explaining the intention to use MHC, younger age and shorter length of stay exhibited a tendency to increase the intention to use MHC. In contrast, Sanchez-Cao et al. [32] have indicated that a longer length of stay is more likely to result in the intention und utilization of MHC. Sanchez-Cao et al. [32] attributed their findings to superior language skills, acculturation within a Western country, and, consequently, a diminished fear of stigmatization. It seems reasonable to posit that the provision of language and cultural mediators by the BetterCare project may facilitate the breakdown of barriers in this area of concern. In general, the findings align with those of previous studies, indicating that socio-demographic characteristics exert minimal influence on the intention or actual utilization of MHC among refugee and immigrant populations [45; 32].

The impact of symptom severity and the degree of impairment caused by the mental illness can exert a considerable influence on intention and utilization of MHC. In alignment with the findings of Lamkaddem et al. [23], our research indicated that elevated symptom severity in the domain of PTSD is associated with increased MHC utilization rates. This result is particularly noteworthy when considered in the context of the fact that only UYRs with clinically relevant PTSS were subjected to analysis. In contrast, other studies have demonstrated that an elevated level of PTSD symptoms is associated with reduced utilization rates [25, 46]. The authors attributed this phenomenon to avoidance behavior and general impairment caused by the symptoms, which impede individuals from seeking help. Support from youth welfare staff and the specific treatment offered in our sample may have facilitated the overcoming of obstacles in this regard. Those affected were not compelled to conduct independent research into treatment options or to engage in the potentially distressing pursuit of free treatment placements. Bean et al. [8] found that a higher number of PTEs led to higher perceived need for professional help. In contrast, there was no significant correlation between PTEs and intention or utilization in the present study. The average number of traumatic events experienced in our sample was high (*M* = 8.1, *SD* = 2.97). It is possible that the Covid-19 pandemic has had an impact on this situation, creating additional challenges for youth welfare workers and psychotherapists in terms of establishing connections and accessing resources. Additionally, Sanchez-Cao et al. [32] found that higher depression symptom severity was associated with lower utilization. In our study, there was no association between depressive symptoms and intention or utilization of MHC. As mentioned above, it is possible that the provision of a specific treatment offer and the existing support from youth welfare staff may have compensated for the effects of any reduced drive that may have been present. Overall, our findings are consistent with Ajzen’s model of planned behavior, which asserts that intention is the primary determinant of behavior, in this case, actual utilization of TF-CBT [36]. In practice, this implies that UYR exhibiting an intention to utilize MHC should be identified and offered an appropriate course of treatment. For UYR who have no intention, the intention should be encouraged, for example through specific psychoeducational interventions.

The present study has several strengths, including the investigation of a difficult-to-reach sample and the examination of utilization within the context of a stepped-care approach for trauma-focused EBT. Furthermore, looking at the data descriptively, almost all UYRs who expressed an intention to seek MHC had at least one session with a psychotherapist. Beside these strengths, several limitations warrant acknowledgment. Firstly, the size of the sample analyzed was relatively small at *n* = 105, which may limit the generalizability of the findings. Secondly, the sample was rather homogeneous with respect to certain variables, such as gender, religion, or country of origin. As a result, the impact of these variables on intention and utilization could not be investigated. Nevertheless, our sample was demographically quite comparable to the refugee population in Germany, as most asylum applications were submitted by Syrian and Afghan male refugees [54]. Thirdly, our sample may have been somewhat selective. This study was conducted with the participation of facilities that were open to the provision of mental health services, aware of the psychological needs of UYRs, and expressed dissatisfaction with the existing treatment options. No conclusions can be drawn about what factors contribute to the use of MHC by UYR who do not have supportive caregivers or who are not participating in a stepped care approach with explicit treatment recommendations. Furthermore, our therapeutic recommendation solely encompassed a trauma-focused intervention. Our findings indicate that elevated PTSD levels are more likely to result in UYRs’ intention to utilize the intervention. This may be due to the fit of the intervention with the symptoms exhibited. It is therefore not possible to generalize the results to interventions with a different treatment focus, e.g. depression, without further investigation. Fourthly, the characteristics of the support provided or the quality of the relationship with caregivers were not subject of investigation. These factors are certainly relevant with regard to the issue at hand and should be given due consideration in future studies. Fifthly, the amount of variance explained in our regression analysis was relatively small. Therefore, there must be other predictor variables that we did not measure. Variables to consider in future research include, for example, the mental health literacy of UYRs, UYRs’ attitudes towards MHC, and the fear of stigmatization [27, 40, 41, 47]. Therefore, according to Ajzen’s model, the influence of UYRs’ attitudes on intention and thus utilization should be examined more closely. In addition, the fit of the model to the representation of the utilization behavior of UYRs cannot be fully confirmed by the statistical methodologies employed in this study. It is imperative that further investigations are conducted, and that mediation analyses or structural equation models are implemented in order to ascertain the model’s suitability in terms of MHC utilization by UYRs. Finally, it should be noted that the present study exclusively focused on utilization of MHC. It is evident that the maintenance of patients in treatment is a crucial element in attaining a sustained reduction in symptoms. It is thus recommended that future studies prioritize the investigation of strategies to prevent premature termination of MHC by UYR. This is a crucial strategy for achieving long-term positive outcomes on both an individual and societal level. In addition, subsequent studies should prioritize the examination of UYRs who articulate an intention yet ultimately do not utilize MHC. The mediating factors in this context are of particular importance; however, their nature remains to be investigated.

In conclusion, this study provides evidence of some variables related to intention and actual utilization of trauma-focused EBT of UYRs. Upon articulating an intention, UYRs in our sample took advantage of treatment offers and attended a minimum of one session with a psychotherapist. As a result of these findings, it is particularly important to strengthen the role of UYRs caregivers (e.g. social workers in CYWS). By equipping caregivers with the necessary tools and knowledge on mental health and respective treatment options, they might be able to support UYR even better in starting an evidence-based treatment. As potential gatekeepers, they could be trained to identify early signs of mental health problems, provide appropriate guidance, and facilitate access to professional help.

To increase the intention to seek help and, consequently, the utilization of MHC, it is essential to also prioritize the mental health literacy of UYRs. Mental health literacy facilitates the capacity to discern self-perceived needs for assistance and to identify suitable professional support [41]. Low mental health literacy represents a pervasive obstacle to the utilization of mental health services among trauma survivors in general [55]. A promising approach to a low-threshold psychoeducational intervention for refugees designed to increase mental health literacy in a culturally sensitive manner is the ‘Tea Garden’ by Mewes et al. [56]. The Tea Garden program is a low-threshold and transdiagnostic intervention. Its objective is to enhance knowledge about mental health problems and available treatment options, and to improve psychological resilience and self-care [56]. In addition to comprehensive screening and treatment recommendations, it may be beneficial to incorporate such low-threshold psychoeducational interventions into UYR stepped-care models. In this way, both structural and individual barriers to utilization of MHC, such as reservations about psychotherapy or fear of stigmatization, could be overcome.

## Electronic supplementary material

Below is the link to the electronic supplementary material.

Supplementary Material 1

## Data Availability

The datasets generated for this study are available upon request from the corresponding author.

## Abbreviations

CYWS: Child and youth welfare system
EBT: Evidence based treatment
PTSD: Posttraumatic stress disorder
PTSS: Posttraumatic stress symptoms
MHC: Mental health care
UYRs: Unaccompanied young refugees
TPB: Theory of planned behavior
