# Supplementary material for: Investigating factors influencing utilization of trauma-focused cognitive behavioral therapy among unaccompanied young refugees: an exploratory analysis
Source: Child Adolesc Psychiatry Ment Health. 2025 Feb 6;19:7. doi: 10.1186/s13034-025-00862-z (PMC11803976; doi:10.1186/s13034-025-00862-z)
Supplement: Supplementary file 1 — Supplementary Material 1 [file 13034_2025_862_MOESM1_ESM.docx]

**Supplement**

**Table S1**

*Sensitivity analyses*

|  |  |  |  |  |  | **95% CI for OR** | |
| --- | --- | --- | --- | --- | --- | --- | --- |
|  | ***B*** | ***SE*** | **Wald** | ***p*** | **OR** | **Lower** | **Upper** |
| UYRs above the clinical Cut-Off for depressive symptoms (a) | | | | | | | |
| Age | -0.22 | 0.21 | 1.02 | .31 | 0.81 | 0.53 | 1.22 |
| Length of stay | -0.57 | 0.34 | 2.71 | .10 | 0.57 | 0.29 | 1.11 |
| CATS-2 | 3.01 | 1.44 | 4.40 | .04 | 20.33 | 1.22 | 339.24 |
| Constant | -5.15 | 5.78 | 0.79 | .37 | 0.01 |  |  |
| UYRs below the clinical Cut-Off for depressive symptoms (b) | | | | | | | |
| Age | -0.43 | 0.54 | 0.63 | .43 | .65 | 0.23 | 1.88 |
| Length of stay | 0.37 | 0.67 | 0.28 | .60 | 1.45 | 0.37 | 5.71 |
| CATS-2 | 4.09 | 4.00 | 1.05 | .31 | 59.66 | 0.02 | 150167.14 |
| Constant | -6.56 | 14.98 | 0.19 | .67 | .00 |  |  |
| UYRs above the clinical Cut-Off for anxiety symptoms (c) | | | | | | | |
| Age | -0.54 | 0.31 | 3.16 | .08 | 0.58 | 0.32 | 1.06 |
| Length of stay | -0.76 | 0.41 | 3.48 | .62 | 0.47 | 0.21 | 1.04 |
| CATS-2 | 3.48 | 1.72 | 4.11 | .04 | 32.56 | 1.12 | 944.62 |
| Constant | -0.96 | 7.54 | 0.02 | .90 | 0.38 |  |  |
| UYRs below the clinical Cut-Off for anxiety symptoms (d) | | | | | | | |
| Age | -0.06 | 0.32 | 0.04 | .84 | 0.94 | 0.51 | 1.75 |
| Length of stay | -0.12 | 0.45 | 0.07 | .79 | 0.89 | 0.37 | 2.16 |
| CATS-2 | 3.20 | 2.24 | 2.04 | .15 | 24.45 | 0.30 | 1967.71 |
| Constant | -9.16 | 8.04 | 1.30 | .26 | 0.00 |  |  |

*Note.* CATS-2 Child and Adolescent Trauma Screen 2, a) *R*^2^ = 0.16 (Cox & Snell), 0.22 (Nagelkerke), Model *χ*^2^ (8) = 3.93, *p* = 0.86, b) *R*^2^ = 0.10 (Cox & Snell), 0.14 (Nagelkerke), Model *χ*^2^ (8) = 4.87, *p* = 0.78, c) *R*^2^ = 0.21 (Cox & Snell), 0.30 (Nagelkerke), Model *χ*^2^ (7) = 9.62, *p* = 0.21, d) *R*^2^ = 0.08 (Cox & Snell), 0.11 (Nagelkerke), Model *χ*^2^ (7) = 14.92, *p* = 0.04
